# Correction: Sarkar et al. The Growing Significance of microRNAs in Osteoporosis. *Cells* 2025, *14*, 1905

**DOI:** 10.3390/cells15141274

**Published:** 2026-07-16

**Authors:** Alika Sarkar, Sana Sarkar, Afreen Anwar, Ji Woong Kim, Jae-Hyuck Shim, Aijaz Ahmad John

**Affiliations:** 1Gene Regulation Section, Laboratory of Molecular Biology and Immunology, National Institute of Aging, Baltimore, MD 21224, USA; alika.sarkar@nih.gov; 2Department of Neurology, The Johns Hopkins University, Baltimore, MD 21205, USA; ssarkar25@jh.edu; 3Department of Biology and Biotechnology, Worcester Polytechnic Institute, 100 Institute Road, Worcester, MA 01609, USA; afreenanwar2712@gmail.com; 4Department of Genetic and Cellular Medicine, University of Massachusetts Chan Medical School, Worcester, MA 01655, USA; jiwoong.kim41@umassmed.edu; 5Horae Gene Therapy Center, University of Massachusetts Chan Medical School, Worcester, MA 01655, USA; 6Li Weibo Institute for Rare Diseases Research, University of Massachusetts Chan Medical School, Worcester, MA 01655, USA

## References

In the original publication [1], some references need to be revised. The details can be found below.

The previous ref. [2] was changed to “Downey, P.A.; Siegel, M.I. Bone biology and the clinical implications for osteoporosis. *Phys. Ther.*
**2006**, *86*, 77–91”. As Downey & Siegel (2006) is a foundational and widely cited review that clearly explains core concepts of bone biology, including osteoblast, osteoclast, and osteocyte function, bone-remodeling dynamics, and the physiological basis of skeletal homeostasis, this reference is very important for helping non-expert readers to understand osteoporosis and how this disease can be reversed by using various disease-modifying agents, including microRNAs.

The ref. [19] “Saag, K.G.; Shane, E.; Boonen, S.; Marín, F.; Donley, D.W.; Taylor, K.A.; Dalsky, G.P.; Marcus, R. Teriparatide or alendronate in glucocorticoid-induced osteoporosis. *N. Engl. J. Med.* **2007**, *357*, 2028–2039.” was moved from ref. [19] to ref. [21].

The ref. [67] “Alwani, A.; Baj-Krzyworzeka, M. MikroRNA jako cel terapeutyczny w chorobach nowotworowych. *Postępy Biochemii* **2021**, *67*, 259–267.” was removed because it was published in Polish and therefore is not accessible to the broader international readership of the journal.

The ref. [92] “Yan, T.-B.; Li, C.; Jiao, G.-J.; Wu, W.-L.; Liu, H.-C. TIMP-1 suppressed by miR-138 participates in endoplasmic reticulum stress-induced osteoblast apoptosis in osteoporosis. *Free Radic. Res.* **2018**, *52*, 223–231.” was removed because the study focuses on miR-138, whereas the corresponding section of our manuscript discusses miR-29a.

The ref. [194] “Shi, C.; Qi, J.; Huang, P.; Jiang, M.; Zhou, Q.; Zhou, H.; Kang, H.; Qian, N.; Yang, Q.; Guo, L. MicroRNA-17/20a inhibits glucocorticoid-induced osteoclast differentiation and function through targeting RANKL expression in osteoblast cells. *Bone* **2014**, *68*, 67–75.” was deleted as the study examines the effects of miR-17/20a on osteoclast differentiation, whereas the relevant paragraph in our manuscript addresses the role of miR-34a. To maintain accuracy and consistency, only references directly related to miR-34a were retained.

Ref. [198] “Huber, J.; Longaker, M.T.; Quarto, N. Circulating and extracellular vesicle-derived microRNAs as biomarkers in bone-related diseases. *Front. Endocrinol.* **2023**, *14*, 1168898” was moved from [198] to [195].

Ref. [197] “Xu, Y.; Li, D.; Zhu, Z.; Li, L.; Jin, Y.; Ma, C.; Zhang, W. miR-27a-3p negatively regulates osteogenic differentiation of MC3T3-E1 preosteoblasts by targeting osterix. *Mol. Med. Rep.* **2020**, *22*, 1717–1726.” and ref. [198] “Sun, L.; Lian, J.X.; Meng, S. MiR-125a-5p promotes osteoclastogenesis by targeting TNFRSF1B. *Cell. Mol. Biol. Lett.* **2019**, *24*, 23.” were removed because they describe biological functions of specific miRNAs (miR-27a-3p and miR-125a-5p), whereas the paragraph focuses on therapeutic technologies for miRNA inhibition and replacement. As they do not align with the content of this section, they have been excluded.

The ref. “Hasanzad, M.; Doabsari, M.H.; Rahbaran, M.; Banihashemi, P.; Fazeli, F.; Ganji, M.; Nameghi, S.M.; Sarhangi, N.; Nikfar, S.; Meybodi, H.R.A. A systematic review of miRNAs as biomarkers in osteoporosis disease. *J. Diabetes Metab. Disord.*
**2021**, *20*, 1391–1406.” was introduced as ref. [198] because it offers a high-quality, systematic evaluation of the current evidence on circulating miRNAs as biomarkers in osteoporosis. It strengthens the manuscript by providing authoritative support for statements regarding diagnostic potential, clinical relevance, and the overall landscape of miRNA-based biomarker research.

The ref. [202] “Kocijan, R.; Weigl, M.; Skalicky, S.; Geiger, E.; Ferguson, J.; Leinfellner, G.; Heimel, P.; Pietschmann, P.; Grillari, J.; Redl, H. MicroRNA levels in bone and blood change during bisphosphonate and teriparatide therapy in an animal model of post-menopausal osteoporosis. *Bone* **2020**, *131*, 115104.” was moved to [213].

The ref. [216] “Kerschan-Schindl, K.; Hackl, M.; Boschitsch, E.; Föger-Samwald, U.; Nägele, O.; Skalicky, S.; Weigl, M.; Grillari, J.; Pietschmann, P. Diagnostic performance of a panel of miRNAs (OsteomiR) for osteoporosis in a cohort of postmenopausal women. *Calcif. Tissue Int.*
**2021**, *108*, 725–737.” was moved to ref. [200].

The ref. “Yavropoulou, M.P.; Anastasilakis, A.D.; Makras, P.; Tsalikakis, D.G.; Grammatiki, M.; Yovos, J.G. Expression of microRNAs that regulate bone turnover in the serum of postmenopausal women with low bone mass and vertebral fractures. *Eur. J. Endocrinol.*
**2017**, *176*, 169–176.” was included as ref. [217] because it is the primary study in which the authors demonstrated that miR-124-3p, miR-2861, miR-21-5p, miR-23a-3p, and miR-29a-3p can serve as potential biomarkers of bone turnover in postmenopausal women with low bone mass and vertebral fractures.

The ref. “Shen, H.; Yao, X.; Li, H.; Li, X.; Zhang, T.; Sun, Q.; Ji, C.; Chen, G. Role of exosomes derived from miR-133b modified MSCs in an experimental rat model of intracerebral hemorrhage. *J. Mol. Neurosci.*
**2018**, *64*, 421–430.” was added as ref. [243] because it provides direct insight into miR-133b, offering a more relevant and focused contribution than the previous reference.

The ref. “Luo, Q.; Guo, D.; Liu, G.; Chen, G.; Hang, M.; Jin, M. Exosomes from MiR-126-overexpressing adscs are therapeutic in relieving acute myocardial ischaemic injury. *Cell. Physiol. Biochem.*
**2018**, *44*, 2105–2116.” was added as ref. [244] because it offers valuable and direct insight into miR-126, making it more relevant to the context.

The ref. [246] “Chen, S.; Tang, Y.; Liu, Y.; Zhang, P.; Lv, L.; Zhang, X.; Jia, L.; Zhou, Y. Exosomes derived from miR-375-overexpressing human adipose mesenchymal stem cells promote bone regeneration. *Cell Prolif.*
**2019**, *52*, e12669.” was moved to [245].

The ref. [247] “Wang, N.; Chen, C.; Yang, D.; Liao, Q.; Luo, H.; Wang, X.; Zhou, F.; Yang, X.; Yang, J.; Zeng, C. Mesenchymal stem cells-derived extracellular vesicles, via miR-210, improve infarcted cardiac function by promotion of angiogenesis. *Biochim. Et Biophys. Acta (BBA)-Mol. Basis Dis.* **2017**, *1863*, 2085–2092.” was removed to ensure accuracy and relevance, as this study focuses on miR-210, which is not discussed in the text.

The ref. [248] “Wen, D.; Peng, Y.; Liu, D.; Weizmann, Y.; Mahato, R.I. Mesenchymal stem cell and derived exosome as small RNA carrier and Immunomodulator to improve islet transplantation. *J. Control. Release* **2016**, *238*, 166–175.” was removed to maintain clarity and precision, as the reference number [245] provides more directly relevant information.

With this correction, the order of some references has been adjusted accordingly.

## Error in Table

In Table 6 under the section “Extracellular vesicle-based delivery systems,” we replaced “mir-193b-enriched exosomes” with “mir-133b-enriched exosomes”. The corrected table is shown below.

## Error in Funding

In funding part, the funding “R01AR086572” was removed due to this publication is not sufficiently related to the scientific aims of the funded project, the corrected funding part was shown below:**Funding:** This project was supported by grants from the NIH/NIAMS (R01AR078230, R21AR084644), the Li Weibo Institute for Rare Diseases Research Pilot Grant Program, Dong-A ST, and the Ministry of Science and ICT-National Research Foundation of Korea.

The authors state that the scientific conclusions are unaffected. This correction was approved by the Academic Editor. The original publication has also been updated.

## Figures and Tables

**Table 6 cells-15-01274-t006:** Delivery strategies to improve miRNA therapeutic stability, specificity, and efficacy.

Non-Virus-Based miRNA Delivery System			
Delivery System	Examples	Advantages	Disadvantages
Cationic lipoplexes	Cationic lipids with hydrophilic heads and hydrophobic tails forming complexes with nucleic acids [230].	Non-immunogenic, easy to manufacture, biocompatible.	Low efficiency and cytotoxicity.
Commercial lipoplexes	Lipofectamine^®^ RNAiMAX, SiPORT™ (Invitrogen, Waltham, MA, USA) [231,232] SilentFect™ (Bio-Rad, Hercules, CA, USA) [233].		
Polyethylene glycol (PEG)-modified liposomes	Cationic lipids conjugated with PEG [234]		
Polymeric delivery systems	Polyethyleneimines (PEIs) [235], low-molecular-weight PEIs [236], PEG/PEI conjugates [237,238], poly (lactic-co-glycolic acid) (PLGA) [239].	Non-immunogenic, transient expression, high packaging capacity.	Low gene-delivery efficiency in vivo, cytotoxicity.
Inorganic compound-based delivery systems	Gold nanoparticles (AuNPs) [240], PEG–gold nanoparticles [241], silica nanoparticles (SiNPs) [242].	High packaging capacity, non-immunogenic.	Low gene-delivery efficiency.
Extracellular vesicle-based delivery systems	miR-133b-enriched exosomes [243], miR-126-enriched exosomes [244], anti-miR-375–enriched BMSC exosomes [245].	High packaging capacity, non-immunogenic, tissue-specific delivery.	Large-scale EV production is challenging; there is a need for biogenesis control, immune profiling, and optimized administration routes.
**Virus-based miRNA delivery systems**			
Retroviral vectors (RVs)	Derived from lipid-enveloped RNA viruses [246] (e.g., Moloney murine leukemia virus, MoMLV) (miR-138 overexpression in murine embryonic fibroblasts) [247].	Stable transgene expression.	High carcinogenic potential due to insertional mutagenesis; cannot transduce non dividing cells.
Lentiviral vectors (LVs)	Lentivirus genus of *Retroviridae* family, including bovine (BIV), feline (FIV), equine (EIAV), simian (SIV), and human (HIV-2) immunodeficiency viruses [248–251].	Stable transgene expression, transduces dividing and non-dividing cells, broader tropism; long-term expression; lower insertional oncogenesis risk.	High chances of random genomic integrations which may cause insertional mutagenesis.
Adeno-Associated Virus (AAV) Vectors	AAV-based systems expressing artificial or therapeutic miRNAs, such as hemagglutinin-specific miRNAs [252] and miR-298 [253], have shown protective effects against influenza and neuromuscular diseases, respectively, in mice. AAV9 mediated suppression of *Shn3* to improve bone phenotype in osteoporotic mice [254].	Low immunogenicity, high transduction efficiency over a wide variety of cells, targeted tissue-specific gene expression based on serotype, infects dividing and non-dividing cells.	Low packaging capacity, production is tedious and expensive, immune activation is possible in large animals/humans; affected by promoter and serotype.
